# Retrospective study of treatment outcomes and complications of cyclocryotherapy in 58 glaucoma-affected dogs from 2018 to 2023

**DOI:** 10.14202/vetworld.2025.606-615

**Published:** 2025-03-18

**Authors:** Chatrawee Tuaktaew, Natthanet Sritrakoon, Winyu Karntip, Nuanwan Rujirekasuwan, Nuttatida Nimitchaiyapong, Burin Nimsuphan

**Affiliations:** 1 Graduate Program in Veterinary Clinical Studies, Faculty of Veterinary Medicine, Kasetsart University, Bangkok 10900, Thailand; 2 Ophthalmology Unit, Kasetsart University Veterinary Teaching Hospital, Bangkok 10900, Thailand; 3 Department of Parasitology, Faculty of Veterinary Medicine, Kasetsart University, Bangkok 10900, Thailand

**Keywords:** cyclocryotherapy, dogs, glaucoma, intraocular pressure, veterinary ophthalmology

## Abstract

**Background and Aim:**

Glaucoma is a neurodegenerative disease characterized by elevated intraocular pressure (IOP) and can lead to irreversible blindness in dogs. Cyclocryotherapy, a cyclodestructive surgical technique, reduces IOP by damaging the ciliary body epithelium to decrease aqueous humor production. Limited data exist on its clinical outcomes and complications in canine patients. This study aimed to evaluate the efficacy and post-operative complications of cyclocryotherapy in dogs with primary and secondary glaucoma.

**Materials and Methods:**

A retrospective analysis was conducted on the medical records of 73 eyes from 58 dogs treated with cyclocryotherapy at Kasetsart University Veterinary Teaching Hospital (2018–2023). The procedure involved applying a double cycle of freezing and thawing using a cryoprobe on 8–10 scleral sites. Evaluations occurred at weeks 1 and 2, then at 1, 3, and 6 months post-operatively. Data collected included IOP, anti-glaucoma medication use, post-operative complications, and vision status. Statistical analyses involved paired t-tests, Chi-square tests, and repeated measures analysis of variance.

**Results:**

Primary glaucoma was present in 40 eyes (54.79%) and secondary glaucoma in 33 eyes (45.21%). Mean pre-operative IOP for primary and secondary glaucoma was 42 ± 36 mmHg and 50.7 ± 14.5 mmHg, respectively, significantly decreasing to 18.3 ± 12.84 mmHg and 14.42 ± 12.06 mmHg at the final follow-up (p < 0.001). The success rate was 83.56%, with 92.30% of eyes preserving vision. Post-operative complications occurred in 50% of cases, primarily conjunctivitis (28.76%). The frequency of anti-glaucoma medication use significantly decreased in both groups (p < 0.001).

**Conclusion:**

Cyclocryotherapy effectively manages canine glaucoma, significantly reducing IOP and medication dependence while preserving vision in most cases. Although complications were noted, they were generally manageable. Prospective studies are recommended to refine treatment protocols and validate these findings.

## INTRODUCTION

Glaucoma comprises a diverse group of progressive disorders characterized by apoptosis of retinal ganglion cells and a distinct optic neuropathy associated with optic disk cupping. In animals, elevated intraocular pressure (IOP) is a critical risk factor for glaucoma, which can lead to irreversible blindness if not managed appropriately [1]. Canine glaucoma is categorized based on its underlying causes (primary, secondary, and congenital glaucoma), gonioscopic characteristics (open, narrow, and closed iridocorneal angle or goniodysgenesis), and disease duration (subacute, acute, and chronic). The non-pigmented ciliary body epithelium produces aqueous humor, which is predominantly eliminated from the anterior segment of the eye through the ciliary cleft. This fluid plays a key role in nourishing and removing metabolic waste from avascular ocular structures such as the lens, posterior cornea, and anterior vitreous. An imbalance between aqueous humor production and drainage can lead to fluid retention in the anterior chamber, resulting in increased IOP [1–3]. Diagnosing glaucoma in dogs involves assessing ocular signs through ophthalmic examinations, tonometry, and gonioscopy. The initial management strategy typically involves topical eye drops containing anti-glaucoma and anti-inflammatory agents to lower IOP and alleviate clinical symptoms. When medical treatments fail to control elevated IOP and clinical signs, surgical interventions become necessary. Surgical approaches to glaucoma treatment are broadly classified into two categories: (1) enhancing aqueous humor drainage through glaucoma filtering surgeries and (2) reducing aqueous humor synthesis by damaging the pars plicata using cyclodestructive techniques [1, 4, 5].

Cyclodestructive techniques, such as cyclocryotherapy and transscleral cyclophotocoagulation, employ mechanical or chemical methods to destroy the ciliary body and ciliary process, offering an alternative treatment approach for canine glaucoma [1, 6, 7]. Studies in human medicine comparing the efficacy of cyclocryotherapy with transscleral cyclophotocoagulation indicate that cyclocryotherapy offers superior IOP reduction and reduces the need for anti-glaucoma medications post-surgery [8]. The procedure involves applying a freezing cryoprobe to the bulbar conjunctiva to ablate the ciliary body epithelium, thereby decreasing aqueous humor production [1, 9, 10]. Cyclocryotherapy typically utilizes liquid and gaseous nitrous oxide or liquid nitrogen as a cryogen, while carbon dioxide, although an option, presents challenges in achieving rapid freezing due to its lower temperature potential [11]. The mechanism of tissue damage involves dehydration, electrolyte imbalance, cellular membrane disruption through crystallization, lipid and protein denaturation, vascular insufficiency, thermal shock, and cell apoptosis [12]. In general, cyclocryotherapy is recommended for cases of visual glaucoma that do not respond to laser cyclophotocoagulation or aqueous humor shunt placement [1]. It is also considered a viable treatment option for end-stage glaucoma, with contraindications including intraocular infections and ocular neoplasia. In human patients, both pediatric and adult, cyclocryotherapy has demonstrated effectiveness in reducing IOP while minimizing complications [13, 14]. Furthermore, cyclocryotherapy has been shown to achieve greater reductions in aqueous humor production compared to transscleral diode cyclophotocoagulation [8, 10].

In Thailand, the rising population of domestic dogs has corresponded with an increase in cases of canine glaucoma. Many of these cases are refractory to anti-glaucoma eye drops, necessitating alternative treatment options such as cyclocryotherapy to control IOP. However, there is a paucity of clinical data regarding the treatment outcomes and complications of cyclocryotherapy in dogs. Therefore, this study aimed to assess the complications and treatment outcomes of cyclocryotherapy in canine glaucoma under clinical conditions.

## MATERIALS AND METHODS

### Ethical approval

The authors declare that the dogs in the study were handled and treated according to the recommendations of the Institutional Animal Care and Use Committee of Kasetsart University, Bangkok, Thailand (ACKU67-VET-030). This study was supervised for animal care and use for scientific research by the principal investigator (National License No. U1-03382-2559).

### Study period and location

The study was conducted from May 2018 to October 2023 at the Ophthalmology Unit, Kasetsart University Veterinary Teaching Hospital, Bangkok, Thailand.

### Study design

This study was retrospective cohort analysis. The main limitations of this study include those inherent to all retrospective clinical data collection.

### Animals

Medical records were collected from the 73 eyes of 58 dogs diagnosed with glaucoma at the Ophthalmology Unit, Kasetsart University Veterinary Teaching Hospital, from May 2018 to June 2023. Anti-glaucoma eye drops were prescribed for all dogs, and their effectiveness was evaluated during follow-up ophthalmic examinations. Cyclocryotherapy was chosen for each dog when the elevated IOP was uncontrolled using anti-glaucoma eye drops. All dogs in the study were prescribed anti-glaucoma eye drops, in which the type and frequency of application depended on the levels of elevated IOP and the type of glaucoma. Anti-glaucoma eye drops included 1% brinzolamide (Alcon, USA), 0.5% timolol (Sangthai Medical Co., Ltd, Thailand), 0.005% latanoprost (Bausch & Lomb, USA), and 0.0015% tafluprost (Santen, Japan). The inclusion criteria for animal recruitment were glaucomatous dogs, which were not restricted to gender, age, breed, and type of glaucoma. There were no exclusion criteria in this study. Clinical data consisted of the presenting date, sex, breed, age, and pre-/post-operative data of the ophthalmic examinations.

### Ophthalmic examinations

Complete ophthalmic examinations were performed on all dogs, including the distant examination and Schirmer’s Tear Test I (STT ophthalmic strips, 32 K. Supply Co., Ltd., Thailand), tonometry (iCare TONOVET, Finland), and fluorescein staining (32 K. Supply Co., Ltd., Thailand), neuro-ophthalmic examination, slit-lamp biomicroscopy (SL-15, Kowa, Japan), and indirect ophthalmoscopy with 20D condensing lenses (Volk, USA). Vision was assessed by the presence of an intact menace response, negotiation through an obstacle course, and observation by the pet owners during pre-operation and each monitoring time after cyclocryotherapy. Additional data, including types of glaucoma (primary versus secondary glaucoma), pre- and post-operative anti-glaucoma eye drops, post-operative complications at each monitoring time, and additional surgical treatments, were collected and analyzed. The underlying causes of secondary glaucoma were also recorded. The ophthalmic examination of each dog was performed by the same veterinary ophthalmologist before and after each follow-up.

### Surgical procedure

All dogs underwent complete physical examination and health check-ups before cyclocryotherapy. The dogs were pre-medicated, intubated, and underwent general anesthesia. Under general anesthesia, cyclocryotherapy was performed as follows: A glaucoma cryoprobe (Cryo-Line, Optikon, Italy) was applied directly to the sclera approximately 5 mm posterior to the limbus. Nitrous oxide was used as a cryogen to achieve freezing temperatures between -60°C and -80°C. A double cycle of freezing and slow thawing, 60 s per cycle, was performed on eight to ten sites to destroy the ciliary body epithelium. Avoidance of the 3- and 9-o’clock positions was necessary to prevent long posterior ciliary blood vessel injury. Systemic non-steroidal anti-inflammatory and topical anti-inflammatory drugs were prescribed for each dog after cyclocryotherapy. Anti-glaucoma eye drops were continued at the same frequency of application as the prescription before cyclocryotherapy.

### Criteria for success and monitoring

There were four variables of treatment outcomes after cyclocryotherapy, including the success rate of treatment, IOP reduction rate, alteration of the number and frequency of anti-glaucoma eye drops, and post-operative complications. The success rate was defined as an acceptable outcome with IOP ≤25 mmHg and the usage of anti-glaucoma eye drops was less than the prescribed eye drops at the pre-operation stage in terms of number and frequency. The reduction rate refers to the declination in IOP from pre-operation until the last follow-up during the monitoring period. The number and frequency of anti-glaucoma eye drops were recorded at each follow-up. Post-operative evaluation and ophthalmic examination were designated as 1 and 2 weeks and 1, 3, and 6 months. Complications were recorded at each follow-up time point by veterinary ophthalmologists.

### Statistical analysis

Descriptive statistics were employed to summarize demographic and clinical data, including the number of affected eyes, patient demographics, and pre- and post-operative IOP values. Continuous variables, for example, IOP and the number of anti-glaucoma eye drops used, were reported as mean ± standard deviation for normally distributed data and as medians with interquartile ranges for non-normally distributed data. Categorical variables are expressed as frequencies and percentages. Comparison of IOP and the number of anti-glaucoma eye drops between pre-operation and each monitoring time point were performed using the Wilcoxon signed-rank test for non-parametric data and Paired t-test for parametric data. The success rates were compared between the primary and secondary glaucoma groups using Chi-square tests. Repeated measure analysis of variance (ANOVA) was performed to assess changes in IOP and anti-glaucoma eye drops use over the follow-up period in the same dogs. A Kaplan–Meier survival analysis was performed to evaluate the time to recurrence of elevated IOP (>25 mmHg) following cyclocryotherapy. Log-rank tests were used to compare survival curves between the primary and secondary glaucoma groups. Analyses were conducted using the Statistical Package for the Social Sciences software (version 22, IBM Corp., Armonk, NY, USA). All statistical tests were two-tailed, and p < 0.05 was applied.

## RESULTS

Seventy-three eyes of 58 dogs were classified as primary glaucoma 40 eyes (54.79%) of 31 dogs and as secondary glaucoma 33 eyes (45.21%) of 27 dogs underwent cyclocryotherapy. The mean age was 8.4 ± 3.0 years (range 2–15 years). The affected eyes were unilateral (43 eyes/43 dogs) and bilateral (30 eyes/15 dogs) glaucomas. Unilateral glaucoma eyes were 23 and 20 of the right eyes and left eyes, respectively. Overall, the numbers of female and male dogs recruited in the study were 37 and 21, respectively. Animal sex in primary glaucoma was found in females (25) and males (15); thus, the female/male ratio was 1.67:1. Shih Tzu is a major breed (14/58, 24.14%) for glaucoma treatment. Moreover, Shih Tzu was the predominant breed that was diagnosed with primary glaucoma in 13/40 eyes (32.5%). The individual data of each dog with primary and secondary glaucoma are shown in Tables 1 and 2, respectively.

**Table 1:** Data of 40 eyes with primary glaucoma and IOP before cyclocryotherapy and at the final follow-up ophthalmic examination.

Patients	Breeds	Age (years)	Vision before cyclocryotherapy	Intraocular pressure (mmHg)	

				Pre-operation	Final follow-up
1	Shih Tzu	12	L	65	18
2	Crossbreed	7	L	42	25
3	Shih Tzu	6	V	54	20
4	Shih Tzu	6	V	57	4
5	Labrador Retriever	9	L	11	16
6	Shih Tzu	6	L	98	13
7	Shih Tzu	10	L	67	3
8	Shih Tzu	11	L	43	17
9	Chow Chow	7	L	68	35
10	Chow Chow	7	L	39	35
11	Shih Tzu	7	V	30	37
12	Crossbreed	10	L	30	9
13	Crossbreed	10	L	36	7
14	Shih Tzu	7	V	60	13
15	Shih Tzu	7	L	57	17
16	Shih Tzu	11	L	52	3
17	Crossbreed	3	L	39	12
18	Crossbreed	8	L	36	15
19	Chihuahua	8	L	9	4
20	Chihuahua	8	L	30	3
21	Pomeranian	12	L	23	3
22	Pomeranian	12	L	26	7
23	Siberian Husky	7	L	23	12
24	Shih Tzu	9	L	19	21
25	Pomeranian	8	L	30	3
26	Shih Tzu	12	L	34	17
27	Crossbreed	5	L	73	40
28	Crossbreed	5	V	63	40
29	Crossbreed	5	V	47	14
30	Crossbreed	5	L	63	9
31	Chihuahua	8	V	36	20
32	Chihuahua	8	L	78	14
33	Shih Tzu	11	L	64	18
34	Siberian Husky	7	L	44	34
35	Labrador Retriever	8	L	25	12
36	Beagle	11	L	73	25
37	Siberian Husky	10	L	43	20
38	Siberian Husky	5	L	64	60
39	Schnauzer	8	L	21	33
40	Crossbreed	8	L	42	24

L=Loss of vision, V=Vision, IOP=Intraocular pressure

**Table 2:** Data of 33 dog eyes with secondary glaucoma and IOP before cyclocryotherapy and at the final follow-up ophthalmic examination.

Patients	Breeds	Age (years)	Vision before cyclocryotherapy	Intraocular pressure (mmHg)	

				Pre-operation	Final follow-up
1	Siberian Husky	6	L	50	30
2	Poodle	12	L	48	3
3	Golden Retriever	11	L	51	3
4	Golden Retriever	11	L	57	19
5	Poodle	15	V	45	7
6	Poodle	15	L	57	3
7	American Cocker Spaniel	10	L	58	5
8	American Cocker Spaniel	10	L	60	4
9	American Cocker Spaniel	2	L	49	8
10	Chihuahua	9	L	42	3
11	Crossbreed	11	L	58	5
12	Poodle	12	L	42	3
13	Shih Tzu	6	L	58	5
14	Crossbreed	11	L	44	23
15	Crossbreed	5	L	50	12
16	Poodle	13	L	11	4
17	Poodle	13	L	72	9
18	Shih Tzu	11	L	47	15
19	American Bully	2	L	46	25
20	Siberian Husky	3	V	68	22
21	Chihuahua	4	L	60	11
22	French Bulldog	5	V	14	16
23	French Bulldog	5	L	44	23
24	French Bulldog	7	V	73	32
25	French Bulldog	7	V	47	36
26	Crossbreed	10	L	33	3
27	Chihuahua	12	L	86	3
28	Poodle	13	L	50	7
29	Shih Tzu	10	L	55	16
30	French Bulldog	8	L	53	21
31	French Bulldog	8	L	46	24
32	Thai Bangkaew	4	L	39	23
33	Alaskan Malamute	6	V	59	53

L=Loss of vision, V=Vision, IOP=Intraocular pressure

From the overall data of the patients in Tables 1 and 2, vision at pre-operative cyclocryotherapy was found in 13 eyes, whereas the remaining vision after cyclocryotherapy was detected in 12 eyes. In primary glaucoma, there were 23/40 eyes (57.5%) of middle to older (age ≥8 years old) glaucoma patients (Table 1) and 17/40 eyes (42.5%) of the dogs aged under 8 years old.

Pre-operative anti-glaucoma eye drops were prescribed by ophthalmologists to treat glaucoma in dogs, including 1% brinzolamide, 0.5% timolol, 0.005% latanoprost, and 0.0015% tafluprost. The most common anti-glaucoma eye drops combined brinzolamide, timolol, and tafluprost (Table 3).

**Table 3:** Number of pre-operative anti-glaucoma eye drops for 73 eyes; the most prescription was a triple anti-glaucoma medication.

Anti-glaucoma eye drops	Number of eyes (%)
Brinzolamide	6 (8.22)
Latanoprost	3 (4.11)
Brinzolamide + tafluprost	10 (13.70)
Brinzolamide + latanoprost	15 (20.55)
Brinzolamide + timolol	5 (6.85)
Brinzolamide + latanoprost + timolol	16 (21.92)
Brinzolamide + tafluprost + timolol	18 (24.65)

The underlying causes of secondary glaucoma were cataract, corneal ulcer, uveitis caused by *Ehrlichia canis* infection, lens-induced uveitis, ocular trauma, and post-cataract surgery (Table 4). Post-cataract surgery was also found to be the major cause in this study.

**Table 4:** Causes of secondary glaucoma in 33 eyes.

Causes	Number of eyes (%)
Cataract	4 (12.12)
Corneal ulcer	2 (6.06)
Uveitis (*Ehrlichia canis* infection)	2 (6.06)
Lens-induced uveitis	6 (18.18)
Ocular trauma	2 (6.06)
Post-cataract surgery	17 (51.52)

**Figure 1:**
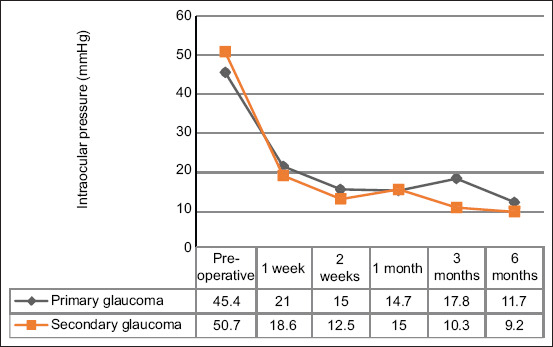
The mean intraocular pressure of 40 and 33 eyes with primary and secondary glaucoma, respectively, before and after undergoing cyclocryotherapy.

The mean IOP values of all dogs at the final follow-up significantly decreased by 64.79% (p < 0.001), as shown in Table 5 and Figure 1. The mean IOP of eyes with primary glaucoma at the final follow-up was significantly reduced by 56.43% (p < 0.001) compared with pre-operation, while the mean IOP of eyes with secondary glaucoma at the final follow-up was also significantly decreased by 71.56% (p < 0.001). The reduction rate of the mean IOP after cyclocryotherapy was higher in patients with secondary glaucoma than in those with primary glaucoma. The success rates were not significantly different between the primary and secondary glaucoma groups (p = 0.557).

**Table 5:** Intraocular pressure and number of anti-glaucoma eye drops applied before and after cyclocryotherapy for primary and secondary glaucoma.

	Primary glaucoma	Secondary glaucoma	Total
Intraocular pressure (mmHg)			
Pre-operation	42.00 ± 36.00	50.70 ± 14.50	47.00 ± 24.50
Final follow-up	18.30 ± 12.84	14.42 ± 12.06	16.55 ± 12.56
p-value	<0.001	<0.001	<0.001
Number of anti-glaucoma eye drops			
Pre-operation	2.5 ± 0.6	2.2 ± 0.7	2.4 ± 0.7
Final follow-up	1.4 ± 1.0	0.9 ± 0.9	1.2 ± 1.0
p-value	<0.001	<0.001	<0.001

**Figure 2:**
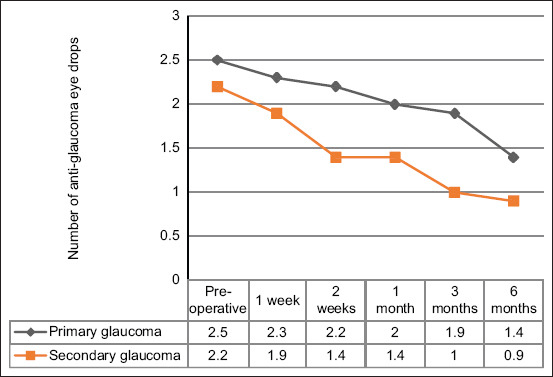
The mean number of topical anti-glaucoma eye drops during follow-up for 40 eyes with primary glaucoma and 33 eyes with secondary glaucoma.

The use of anti-glaucoma eye drops following cyclocryotherapy was significantly decreased (p < 0.001) in both eyes with primary and secondary glaucoma, as shown in Table 5 and Figure 2. The anti-glaucoma medication was discontinued in 17 eyes of 14 dogs within 6 months of the monitoring period after cyclocryotherapy. Overall, the success rate with or without medications in this study was 83.56%, which is defined as an acceptable outcome with IOP 25 mmHg and lowering of both the number and frequency of anti-glaucoma eye drops. In the final follow-up, IOPs of ≤25 mmHg were detected in 32 eyes with primary glaucoma (80%) and 29 eyes with secondary glaucoma (87.88%).

**Figure 3:**
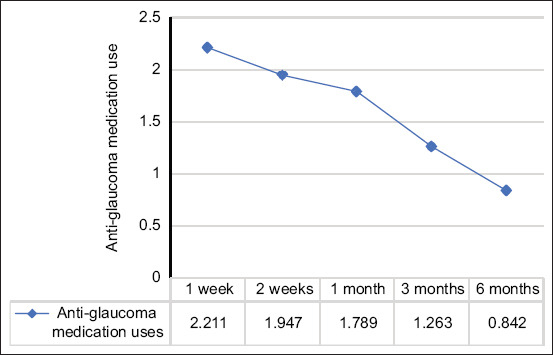
Repeated measures analysis of variance of alteration use of anti-glaucoma medications over the follow-up periods, p < 0.001.

**Figure 4:**
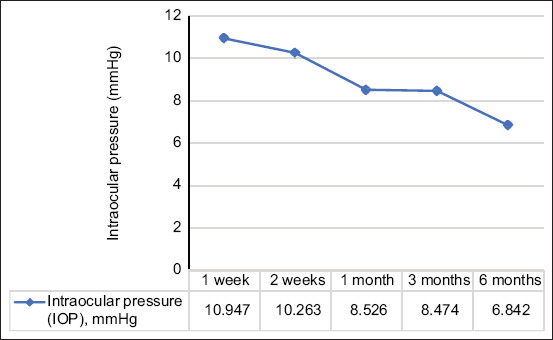
Repeated measures analysis of variance of changes in intraocular pressure over the follow-up period (p = 0.088).

Results of the repeated measures ANOVA show that anti-glaucoma eye drops (Figure 3) uses were significantly decreased with p < 0.001, which were found in 3 and 6 months, whereas the declinations of IOP (Figure 4) following cyclocryotherapy were not significantly different with p = 0.088 over the follow-up periods in the same dog (n = 19).

The success rates after cyclocryotherapy for glaucoma treatment in all brachycephalic and mesocephalic breeds (Tables 1 and 2) were 84.36% and 79.41%, respectively. In patients with primary glaucoma, the success rate of brachycephalic breeds (84.21%) was higher than that of mesocephalic breeds (76.19%), whereas the success rates of brachycephalic and mesocephalic breeds in patients with secondary glaucoma were not different (84.62%).

The mean IOP at each observation time point gradually but significantly declined to acceptable levels in both primary and secondary glaucoma, as shown in Figure 1.

There was no difference in survival time to estimate the recurrence of elevated IOP (>25 mmHg) following cyclocryotherapy in both primary and secondary glaucoma groups with median = 4 months. Twelve eyes did not respond to cyclocryotherapy for decreased IOP, which were composed of eight and four eyes of primary and secondary glaucoma, respectively. Seven eyes of primary glaucoma proceeded to additional treatments, including five and two eyes that underwent enucleation and repeating cyclocryotherapy, respectively. The last eye with primary glaucoma did not receive any additional treatment because the pet owner refused further surgery. The IOP of patients with primary glaucoma who received repeated cyclocryotherapy gradually decreased within 1 month. In cases of secondary glaucoma, three eyes were submitted for repeated cyclocryotherapy, in which two eyes suffered from post-operative glaucoma and the other one eye from *E. canis* infection. Regarding the IOP level of patients with secondary glaucoma who were repeating cyclocryotherapy, one eye responded to treatment, eventually becoming phthisis bulbi, and the other two eyes failed following treatment and underwent enucleation. In addition, one eye with secondary glaucoma suffered from lens-induced uveitis and underwent transscleral cyclophotocoagulation.

Post-operative complications were detected in 29 dogs (50%), as listed in Table 6, while the other 29 dogs did not show any ocular signs of complications. The most common post-operative complication was conjunctivitis (21/73 eyes, 28.76%), which was predominantly found among the other five post-operative complications. Six eyes with corneal ulcers were found in two buphthalmic eyes and four non-buphthalmic eyes. In addition, uveitis and hyphema were detected in 10 (10/40, 25%) and 2 (2/40, 5%) eyes with primary glaucoma, respectively.

**Table 6:** Ocular disorders of post-operative complications after cyclocryotherapy in 73 eyes.

Complications	Number of eyes (%)
Conjunctivitis	21 (28.76)
Chemosis	7 (9.59)
Corneal ulcer	6 (8.22)
Ocular hypotension	7 (9.59)
Phthisis bulbi	8 (10.96)
Third eyelid inflammation	1 (1.37)

Before cyclocryotherapy, there were 13 eyes with vision (Tables 1 and 2). However, after cyclocryotherapy, 12 eyes were considered to have remaining vision (92.30%), whereas only one eye (7.7%) experienced irreversible blindness. Sixty eyes were defined as those with blindness on pre-operation.

In addition, keratoconjunctivitis sicca (KCS) was also found in the follow-up examination in 5 eyes of 4 dogs (4/27 dogs, 14.81%) of whom KCS had never been diagnosed in these 27 dogs. The STT I values were 6, 7, 12, 12, and 13 mm, respectively, which were measured after cyclocryotherapy on days 10–14. These dogs were started on KCS treatment with artificial tears and cyclosporine. Transient acute elevation of IOP (IOP spike) was also detected in three eyes of primary glaucoma patients within 24–72 h after cyclocryotherapy. However, no IOP spike was detected in eyes with secondary glaucoma.

## DISCUSSION

Glaucoma is a chronic, progressive disease that often leads to irreversible vision loss. Cyclocryotherapy has been used as the treatment of choice for glaucoma since 1950 [15]. Cyclocryotherapy is a cyclodestructive technique that attempts to decrease aqueous humor production and ultimately lowers IOP. The parameters indicating successful treatment of glaucoma in dogs in this study included IOP, the number of anti-glaucoma eye drops required after cyclocryotherapy at each follow-up time point, and post-operative complications.

Data were collected from 73 eyes of 58 dogs that underwent cyclocryotherapy during the 6-month post-operative period. The proportion of females to males with affected eyes with primary glaucoma was 1.67:1, consistent with other studies. The ratios in North America and South Korea were 1.5:1 and 1.3:1, respectively [16, 17]. Due to the iridocorneal angle morphology, the angle opening distance in female dogs is significantly smaller than in male dogs [18]. This anatomical difference makes female dogs more susceptible to primary angle-closure glaucoma. In addition, the anterior chamber depth is another important parameter for glaucoma evaluation. Each millimeter decrease in anterior chamber depth is associated with a 2.6-fold increased risk of angle closure [1, 18].

The Shih Tzu breed was the most commonly associated with primary glaucoma. The primary glaucoma characteristic in Shih Tzu dogs is a closed-iridocorneal angle. This finding is similar to previous studies in Korea and Japan, showing incidences of 36.45% and 11.62%, respectively, indicating that Shih Tzu dogs are a major breed for primary glaucoma. However, the Shiba Inu was also identified as a major breed with a higher prevalence than Shih Tzu in primary glaucoma cases [1, 16, 17, 19]. The popularity of dog breeds varies by region and country, which affects the incidence of primary glaucoma in each location.

The factors influencing the success rate of cyclocryotherapy in humans include the cause of glaucoma, the thickness of the sclera (which affects the speed and depth of freezing), and the age of the patient (which influences the efficiency of blood supply to the ciliary processes) [20]. In this study, cyclocryotherapy was more effective for treating secondary glaucoma than primary glaucoma. Unfortunately, there are no reports comparing cyclocryotherapy protocols for different scleral thicknesses in veterinary medicine. In a human study, the freezing time for children’s eyes was 30–45 s, shorter than for adults’ eyes due to the thinner sclera and proximity of the lens [20]. In addition, brachycephalic breeds have significantly thinner eyeballs at the level of the ciliary body and fewer ciliary processes than mesocephalic breeds, potentially affecting the effectiveness of cyclocryotherapy [21]. The success rate for brachycephalic breeds (84.36%) was higher than for mesocephalic breeds (79.41%) due to ocular structural differences.

The most common cause of secondary glaucoma in this study was post-cataract surgery, likely because the veterinary teaching hospital acts as a referral center for ophthalmology. In general, post-operative ocular hypertension is characterized by an acute, transient increase in IOP of >25–27 mmHg between 2 and 48 h after surgery. If post-operative ocular hypertension is not controlled within the normal range, it can develop into post-operative glaucoma. Previous studies reported by an average incidence of post-operative ocular hypertension of 35% [1], while this study found a higher incidence of 51.52%. Potential causes of post-operative ocular hypertension include inflammation, retention of ophthalmic viscosurgical devices, lenticular particle retention, hyphema, iris pigment release, and rapid aqueous humor formation in combination with watertight wound closure [22]. Histological changes after intraocular surgery, including decreased ciliary cleft cross-sectional surface area and width, as well as alterations and swelling in the trabecular meshwork, have also been reported [23]. Factors associated with post-operative ocular hypertension following phacoemulsification include breed, advanced age, diabetes, hypermature cataract stage, prolonged phacoemulsification, intraocular lens placement, high-viscosity ophthalmic surgical devices, pre-operative lens-induced uveitis, post-operative uveitis, intraoperative hemorrhage, and retinal abnormalities [1, 22–24]. In this study, older age, diabetes, and hypermature cataracts were potential contributing factors.

The treatment outcomes suggest that cyclocryotherapy is an effective treatment for glaucoma in dogs. The overall post-operative IOP at each monitoring period significantly decreased compared with the pre-operative IOP (p < 0.001). The IOP at the final follow-up for both primary and secondary glaucoma was also significantly reduced (p < 0.001). The IOP reduction rates for primary and secondary glaucoma increased progressively with follow-up time. The decrease in the number of anti-glaucoma eye drops was statistically significant in both primary and secondary glaucoma groups. At the final observation, 23.29% of eyes, all considered pre-operative blindness, discontinued anti-glaucoma eye drops, which is higher than the 3% reported in human studies [25].

In this study, nine eyes with pre-operative IOP ≤25 mmHg were submitted for cyclocryotherapy due to the maximum frequency and number of eye drops required. There were twelve eyes in which IOPs were >25 mmHg at the final follow-up. Five eyes underwent repeated cyclocryotherapy, in which three eyes showed IOP reduction to <25 mmHg within 2–4 weeks, one eye developed an atrophic globe. The other two eyes did not achieve controlled IOP and required enucleation. In humans, applying the cryoprobe to six quadrants and the 3- and 9-o’clock positions improved cyclocryotherapy effectiveness, achieving a success rate of 90% [13].

The present study found 9.59% and 10.96% of ocular hypotony and phthisis bulbi, respectively. In general, the factors that cause ocular hypotony are the number of freezing points, freezing time, and lower temperature which the freezing temperature is the major factor causing ocular hypotony [15, 26, 27]. The freezing temperature forms the intracellular crystallization, causes severe necrosis of the ciliary body resulting in phthisis bulbi. The excessive destruction from temperature and duration of the procedure are also the etiologies of phthisis bulbi [26, 28]. Cyclocryotherapy in humans was also reported as a cause of phthisis bulbi with an incidence of 0.9%–13.1% in secondary and congenital glaucoma, which is similar to the present study in canine patients [29].

Notably, cataract formation was not observed within 6 months post-cyclocryotherapy in this study. A human study reported that cataract formation occurred in patients who underwent cataract extraction within 5–15 months after cyclocryotherapy [30]. The incidence of cataract formation in human studies was 20%–31% [31, 32].

The post-operative KCS rate was 14.81%, lower than the 44% reported in dogs treated with micropulse transscleral cyclophotocoagulation [33]. The mechanical and thermal stimulations from cyclodestructive techniques can lead to nerve deformation and ischemia, resulting in corneal nerve damage due to ciliary body destruction, ultimately causing corneal hypoesthesia [33–35]. Corneal nerves play a critical role in inducing the blink reflex, promoting ocular surface protection, facilitating wound healing, and enhancing tear production and secretion through autonomic nerves at the lacrimal glands [33, 35]. In this study, no corneal ulceration was observed in post-operative KCS patients.

However, the retrospective nature of this study has inherent limitations. There were several confounding factors, including potential biases in data collection and inconsistent follow-up. The absence of a control group treated with alternative methods, for example, laser cyclophotocoagulation, restricts direct comparative analysis. In addition, the follow-up period may not comprehensively capture long-term complications or recurrences.

Future prospective studies with larger and more diverse cohorts are needed to validate these findings and standardize cyclocryotherapy protocols across veterinary practices. Comparative studies with alternative cyclodestructive techniques, for example, transscleral cyclophotocoagulation, could further delineate the optimal therapeutic approach. Investigating the long-term impact of cyclocryotherapy on visual preservation, quality of life, and the progression of glaucoma in dogs will provide a more holistic understanding of its clinical utility. Finally, advancements in cryogenic technology could enhance the precision and safety of this procedure, warranting further exploration.

## CONCLUSION

This study demonstrates that cyclocryotherapy is a highly effective and safe treatment modality for managing both primary and secondary glaucoma in dogs. The procedure resulted in a significant reduction of IOP from pre-operative values of 42 ± 36 mmHg and 50.7 ± 14.5 mmHg in primary and secondary glaucoma, respectively, to 18.3 ± 12.84 mmHg and 14.42 ± 12.06 mmHg at the final follow-up (p < 0.001). The success rate of 83.56%, coupled with vision preservation in 92.30% of eyes with pre-operative vision, underscores the clinical utility of cyclocryotherapy. In addition, the significant reduction in the use of anti-glaucoma medications post-operatively highlights its potential to improve the long-term management of canine glaucoma. Despite a 50% incidence of post-operative complications, the majority, including conjunctivitis (28.76%), were manageable with standard clinical care.

The primary strength of this study lies in its comprehensive evaluation of a large cohort of canine patients over an extended follow-up period. The inclusion of both primary and secondary glaucoma cases provides valuable insights into the efficacy of cyclocryotherapy across different etiologies. Moreover, the use of robust statistical analyses, including paired t-tests, Chi-square tests, and repeated measures ANOVA, reinforces the validity of the findings.

However, the study’s retrospective design introduces inherent limitations, including potential biases in data collection and the absence of a control group treated with alternative methods. The variability in follow-up duration may also influence the assessment of long-term complications and treatment efficacy. Furthermore, the study’s focus on a single veterinary teaching hospital may limit the generalizability of the results to broader clinical settings.

Future research should adopt a prospective, multicenter design with standardized treatment protocols to validate these findings and refine clinical guidelines for cyclocryotherapy in veterinary ophthalmology. Comparative studies with alternative cyclodestructive techniques, such as transscleral cyclophotocoagulation, are warranted to delineate optimal treatment approaches. In addition, exploring advancements in cryogenic technology may enhance procedural precision, reduce complications, and improve long-term outcomes. Ultimately, these efforts could establish cyclocryotherapy as a cornerstone treatment in the management of canine glaucoma, contributing to improved quality of life and visual preservation in affected dogs.

## AUTHORS’ CONTRIBUTIONS

CT, NS, and BN: Conceptualized and designed the study. NS, WK, and BN: Performed cyclocryotherapy and post-operative evaluations. CT, NR, and NN: Data collection. CT and BN: Drafted manuscripts and performed data validation. CT: Statistical analysis. BN: Supervised the study. All authors have read and approved the final manuscript.
